# Study the Formation Process of Cuboid Microprotrusion by Glass Molding Process †

**DOI:** 10.3390/mi8030066

**Published:** 2017-02-23

**Authors:** Tao Wang, Tianfeng Zhou, Jing Chen, Lu Song

**Affiliations:** 1 National Key Laboratory of Science and Technology on Micro/Nano Fabrication, Peking University, Beijing 100871, China; t.wang2010@pku.edu.cn (T.W.); l.song@pku.edu.cn (L.S.); 2 Key laboratory of Fundamental Science for Advanced Machining, Beijing Institute of Technology, Beijing 100081, China; zhoutf@bit.edu.cn

**Keywords:** glass molding process, finite element modelling, microprotrusion

## Abstract

This paper investigates the formation process of a typical microstructure in the glass microfluidic chip, i.e., cuboid microprotrusion, by the soda-lime glass molding process (GMP). The finite element models on the platform Abaqus/Standard were established for simulating the glass molding process. The glass viscoelasticity at pressing temperature was described by the General Maxwell model. The influence of the temperature, aspect ratio and side wall angle on the replication ratio was investigated, and the corresponding predicted molded profiles were demonstrated as well. The established simulation model was verified by experimental results eventually. It could provide a fundamental experience for optimizing glass molding parameters to fabricate microstructures on glass chips.

## 1. Introduction

Compared with polymer microfluidic devices, devices made by glass are proven to be superior in applications in the biology and chemistry fields due to its high transparency, chemical stability and good biocompatibility [1]. In order to achieve the widespread application of glass microfluidic chips, many researchers have been exploring the field of microstructure fabrication techniques on glass, and the conventional approaches can be classified into four categories: wet etching, dry etching, laser processing and mechanical processing. Chen et al. [2] utilized the wet etching method to fabricate a microstructure in soda-lime glass, and a smooth surface microchannel with a depth of more than 110 µm was achieved after 2 h of etching. However, wet etching is limited to being isotropic and is difficult in high-precision dimension reproducibility due to undercutting. In addition, a metal or Si layer is usually required as the etch mask. Park et al. [3] presented a deep reactive ion etching (DRIE) process of the borosilicate glass using the SF_6_ and SF_6_/Ar inductively coupled plasmas; the influence of the etching rate by various process parameters such as the gas chemistry, the gas flow ratio, the top electrode power, and the bottom electrode power was studied. However, the dry etching method suffered from a low etching rate and low etching selectivity between the glass and photoresists, and the cost was relatively high. Nieto et al. [4] developed a laser-based technique for fabricating microfluidic microchips on soda-lime glass substrates, and the manufactured chips were tested with tumor cells (Hec 1A) after being functionalized with an epithelial cell adhesion molecule (EpCAM) antibody coating. However, the laser method often suffered from microcracks and other collateral damage. Lin et al. [5] used an ultra-precision machining method to fabricate cross-junction channels (14 µm wide and 28 µm deep) with a three dimensional (3D) triangle cross-section. However, the minimum size of the mechanical workability depends on the tool shape, and it is hard to achieve a tool diameter smaller than 50 μm. In addition, it usually suffered from severe tool wear.

As an effective high-volume and net-shape fabrication method, precision glass molding process technology has been proven to be a potential approach for fabricating microstructures on glass [6] in the optical application field. It is widely utilized to fabricate aspherical glass lenses and periodic microstructures with different pitch sizes, such as Fresnel lenses [7]. Sharp-angled cross-sections and curve surface structures were investigated by experiments and the finite element method (FEM) [8,9,10,11], and there are some papers about developing a glass constitutive model [12,13] during the molding process as well.

In terms of cuboid protrusion on microfluidic glass chips, it is an important structure for microscale filters. Chen et al. developed weir-type crossflow filtration microchips for the separation and collection of white blood cells (WBCs) and red blood cells (RBCs) in parallel [14]. Ji et al. designed a weir in microfilter chips, and imposed a cutoff size of 3.5 μm to selectively trap WBCs [15,16]. It is worth pointing out that they are all silicon-based microfilters, and the fabrication of protrusions on microfluidic glass chips has rarely been reported before. Since molding approaches have many advantages compared to conventional fabricating methods, e.g., high efficiency, low cost and good surface quality, exploring the molding of cuboid microprotrusions on glass can create a solid foundation for the widespread application of glass microfluidic chips.

This paper aims to understand the formation process of cuboid microprotrusions by glass molding process (GMP). First, a simulation model for GMP was established, which considered glass viscoelasticity, and the deformation process was analyzed; then the influence of the pressing temperature, aspect ratio (groove height/width) and side wall angle were studied based on the established models. Finally, the experimental results were presented, and the corresponding simulation models were verified quantitatively. The research could provide fundamental knowledge for optimizing molding parameters.

## 2. Experiment Setup

**Figure 1:**
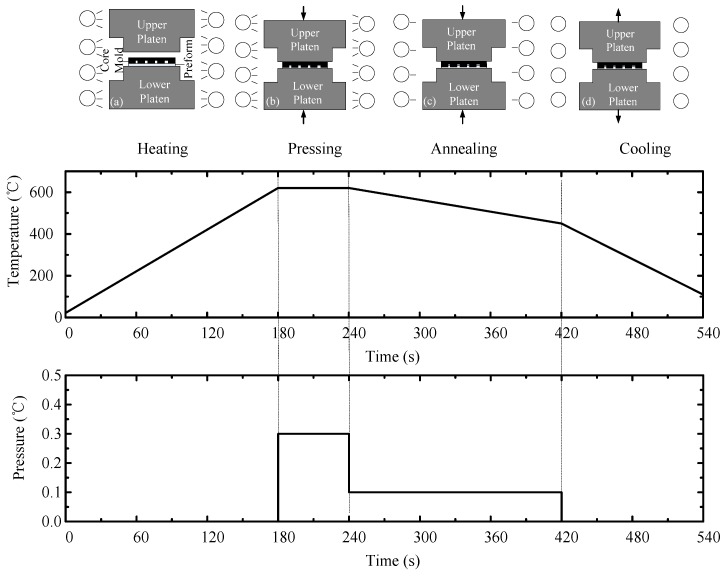
The typical evolution of temperature and pressure during glass molding process (GMP) cycle.

All test glass were molded on the ultraprecision glass molding machine, PFLF7-60A (SYS Corp., Osaka, Japan), which can operate between 20 and 750 °C. The glass molding process can be schematically illustrated in Figure 1. Before experiment, nitrogen gas was utilized to purge the air to protect the molds from oxidation at high temperature. In GMP, the mold and the platens are heated by infrared lamp to above 50–100 °C of glass transition temperature (*T*_g_) for about 180 s. Since glass is transparent in infrared light, it cannot be directly heated by the infrared lamp, but instead, has to be indirectly heated by the core mold and the surrounding gas. Then, pressing is performed to form the microstructure on the glass for 60 s. After that, the annealing is conducted to release the internal stress for 180 s, and in the meantime, small pressure is kept to facilitate the shape transferability. Finally, the molded glass preform is fast cooled to room temperature by water-cooling for 120 s. After demolding, the microstructure on the glass is obtained. The typical evolution of temperature and pressure during GMP cycle is shown in Figure 1.

As for the glass preform material, since the soda-lime glass slides have been used by biomedical researchers to conduct experiments for decades, and it is inexpensive which is beneficial to reduce the whole fabrication cost, it is used in the research as glass preform. The upper and lower platens are made of tungsten carbide (WC) due to its high stiffness and no adhesion characteristics.

**Figure 2:**
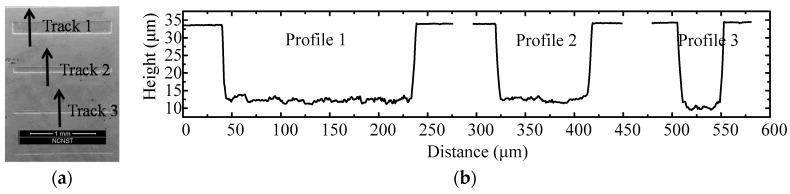
The morphology of the core mold. (**a**) Scanning electron microscopic (SEM) photo; (**b**) profiles along the track.

**Figure 3:**
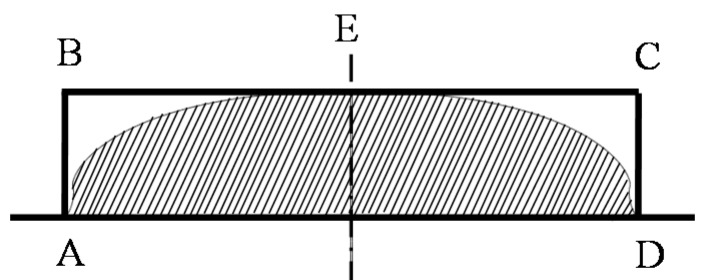
Schematic diagram of the filling ratio (dashed area divided by *S*_ABCD_).

The main morphology of the core mold for swell molding is shown in Figure 2. It is fabricated by inductively coupled plasma (ICP) etching technology. Figure 2a is the scanning electron microscopic (SEM) photo of the groove area on the core mold. In order to demonstrate more details about the groove, the section profile of the second groove is extracted by VK-X200 3D Laser Scanning Microscope (Keyence Co., Ltd., Osaka, Japan ) and shown in Figure 2b, it indicates that the top area is smooth (less than 5 nm), the bottom surface is relative rough. The width of the three grooves are 200, 100 and 50 μm, and all their depths are around 22 μm. In addition, the groove wall is not critically vertical which can make the demolding process easy. The morphology of molded glass profile was obtain by Olympus Lext OLS4100 (Olympus Corp., Tokyo, Japan). In predicted results, the filling ratio is defined by dividing the dash area *S*_AED_ (molded microstructures) by the area of the rectangle area *S*_ABCD_ (the mold), as shown in the Figure 3. The aspect ratio of the mold *r*_a_ is defined by *L*_AB_/*L*_BC_, and the side wall angle θ is defined by ∠BAD.

## 3. Modeling Process

All the simulation models were established in the commercial finite element software Abaqus/Standard. Since the molding speed is relatively slow compared to other machining processes, the GMP is regarded as a quasi-static process. The GMP consists of four stages, i.e., heating, pressing, annealing and cooling. In order to predict the accurate molded shape, the whole GMP should be simulated in a row. However, it is still a challenge to simulate the entire model of a millimeter-scale object surrounded by micro-/submicrometer-scale structures. In this paper, only the pressing stage was simulated for simplicity, and all the molds and preforms were assumed to maintain a constant temperature, which is tens of degrees centigrade above its transition temperature.

**Figure 4:**
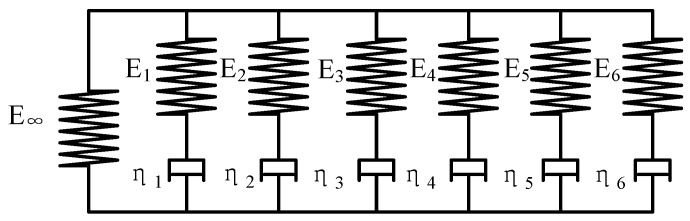
The schematic diagram of Generalized Maxwell model.

At the pressing condition, the preform demonstrated high viscoelasticity, which is a time-dependent response of a material to stress or strain, and they are characterized by creep and stress relaxation behavior. Since the Generalized Maxwell model is proved to be superior to other constitutive models, such as the simple Maxwell model, Kelvin model and Burgers model, it is used to describe creep and stress relaxation in the viscoelastic deformation of glass in the pressing process [17]. In this paper, a six-pair Generalized Maxwell model is used as the constitutive model, and is shown in Figure 4.

The time-dependent response is characterized by the deviatoric terms, as shown below:

(1) $$\sigma \left(t\right)=\int_{0}^{t}G\left(t-\tau \right)\frac{d_{\varepsilon }}{d_{\tau }}d_{\tau }$$

The above integral is evaluated for current time *t* on the basis of past time τ. *G*(*t* − τ) is not a constant value, and it can be represented by a Prony series, as shown below:

(2) $$G\left(t\right)=G_{0}\sum_{i=1}^{n}\omega_{i}e^{-\frac{t}{\lambda_{i}}}$$

where ω_i_ is the relative moduli, and λ_i_ is the reduced time used to describe the shift time due to temperature [17].

The effect of the thermo-rheological simplicity (TRS) is described by the William–Landel–Ferry (WLF) shift function, as shown below:

(3) $$\log\left(A\left(T\left(\tau \right)\right)\right)=\frac{c_{1}\left(T-T_{\tau }\right)}{c_{2}+T-T_{\tau }}$$

where *T*_τ_ is the glass transition temperature, and *c*_1_ and *c*_2_ are the material constants.

**Figure 5:**
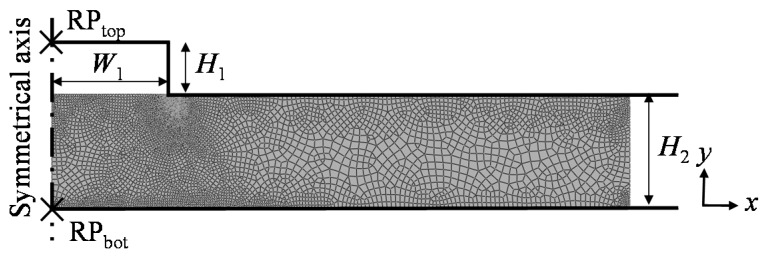
Two dimensional (2D) simulation model for GMP of rectangle microstructure. RP_top_ and RP_bot_ are the coupling constraint with the top and bottom model, respectively.

In the simulation, the properties of the soda-lime preform are listed in Table 1 [18]. The parameters of the Generalized Maxwell model of soda-lime glass are listed in Table 2 [19]. A typical microstructure is molded in Abaqus/Standard. Figure 5 shows the two dimensional (2D) simulation model of GMP for a rectangle microstructure. The bottom model is fixed, and the pressing process is achieved by adding a force boundary condition on the RP_top_ which is a coupling constraint with the top model. In order to facilitate the simulation convergence, the shape bottom corner of the top model is rounded with a radius of 0.5 µm, while the sharp angle of the valley is kept as the actual size. *H*_1_ = 22 μm is set to conform to the actual groove depth on the core mold. The top and bottom molds are both modeled as rigid objects. Since each microstructure is symmetrical, a half model is built for simplicity. In order to alleviate the mesh distortion during deformation, the meshes around the bottom corner of the top model are densified. Friction interactions between the glass and the molds are both modeled by the Coulomb friction model, and the friction coefficient *f* is fixed at 0.1. According to [20], the appropriate model temperature is 620 °C; the range of temperature studied in the simulation was between 620 and 660 °C. Since the normal maximum stress was below 10 MPa in the majority of glass simulation reports, a constant force was set in the simulation to guarantee that.

## 4. Simulation Results and Discussion

### 4.1. Typical Stress Distribution in Molding Process

**Figure 6:**
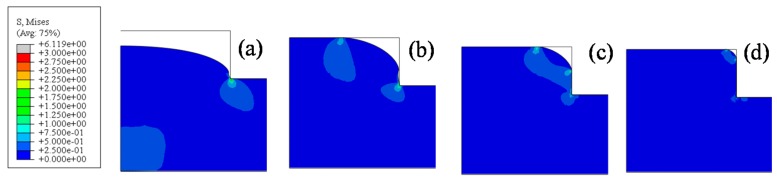
Stress distribution at 640 °C (**a**) 40 s, (**b**) 80 s, (**c**) 100 s and (**d**) 140 s.

Due to the influence of creep characteristics, for a given force and temperature, the filling shape varies with the increasing holding time, as shown in Figure 6. When the holding time was 40 s, the maximum stress location was around the bottom corner of the top mold, and a swell was successfully molded, as shown in Figure 6a. When it increased to 60 s, the swell could touch the top ceiling of the rectangle top mold, and another high-stress location was generated at the end of the contact area, as shown in Figure 6b. When it increased to 100 s, the high deformation of the glass by the squeeze of the mold made it contact with the side wall of the mold, and the third high-stress area was formed, as shown in Figure 6c. When the time increased further, the high-stress areas near both the ceiling and side wall gradually approached to the right corner, and became one, as shown in Figure 6d. The maximum stress in the four movements was around 6.19 MPa.

### 4.2. Influence of Temperature T

**Figure 7:**
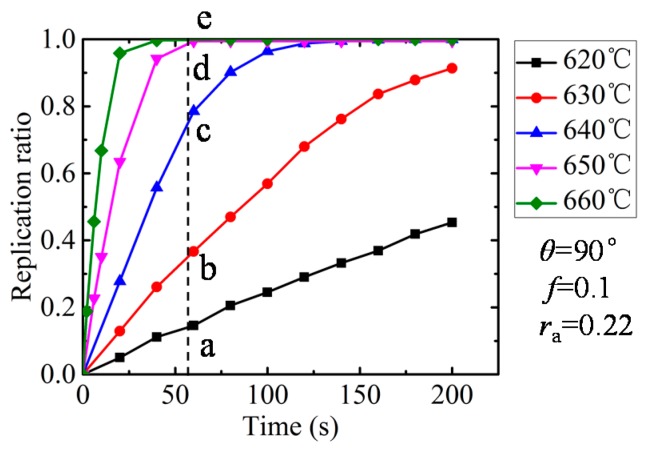
Influence of temperature on replication ratio.

**Figure 8:**
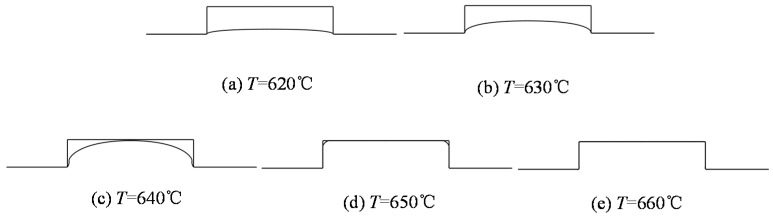
Molded profiles by simulation at different temperature (θ = 90°, *r*_a_ = 0.22). (**a**) *T* = 620 °C, (**b**) *T* = 630 °C, (**c**) *T* = 640 °C, (**d**) *T* = 650 °C and (**e**) *T* = 660 °C.

The influence of the filling ratio is shown in Figure 7. It is evident that the replication ratio increased with the pressing time due to the glass characteristic of stress relaxation and creep under the high-temperature molding process. The partial filling ratios were obtained under the investigated holding time range when the temperature was 620 and 630 °C, while when temperature increased above 640 °C, the replication ratio rose dramatically and achieved complete replication in a short time. The higher temperature, the shorter the hold time required. It is worth pointing out that high temperature can shorten the holding time; in the meantime, it increased the heating time and the risk of adhesion between the mold and glass. Therefore, in practice, an appropriate holding time and temperature should be chosen in GMP. In order to provide more details about molded morphologies, the molded profiles of the simulation at 60 s were extracted and are shown in Figure 8 correspondingly.

### 4.3. Influence of Aspect Ratio r_a_

**Figure 9:**
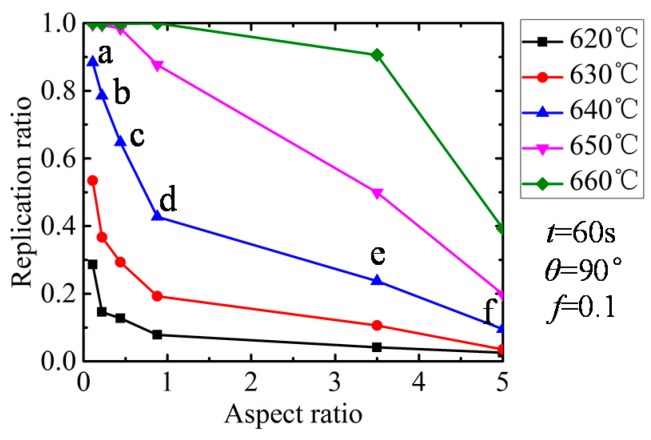
Influence of aspect ratio on replication ratio.

**Figure 10:**
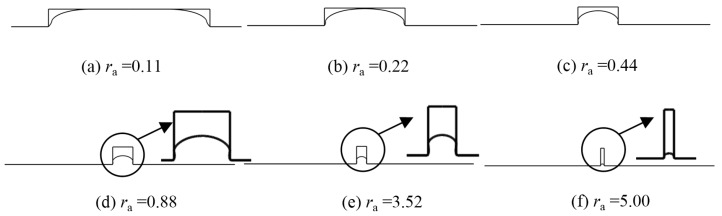
Molded profiles by simulation at different aspect ratios (*t* = 60 s, θ = 90°). (**a**) *r*_a_ = 0.11, (**b**) *r*_a_ = 0.22, (**c**) *r*_a_ = 0.44, (**d**) *r*_a_= 0.88, (**e**) *r*_a_ = 3.52 and (**f**) *r*_a_ = 5.00.

The influence of the replication ratio is shown in Figure 9. All the results were extracted at a holding time of 60 s. It is obvious that the replication increases with the pressing temperature, while it drops with the aspect ratio. When the aspect ratio increased from 0.22 to 1.00, it fell dramatically, and when the aspect ratio increased further, a moderate drop was witnessed. Since glass can fill the mold curve completely at 640 and 650 °C when the aspect ratio is below 0.44, the pattern is not obvious for the two high-temperature curves. The main reason is that the viscoelastic glass tends to flow in the place of small resistance. When the aspect ratio is high, the glass flowing inside is supposed to be confronted with high resistance, thereby lowering the replication ratio. The molded profiles by simulation at the temperature of 640 °C were extracted and are shown in Figure 10 correspondingly. In order to provide more details, the molded areas in Figure 10d,e are enlarged. It is obvious that the higher the aspect ratio, the smaller the replication ratio.

### 4.4. Influence of Side Wall Angle θ

**Figure 11:**
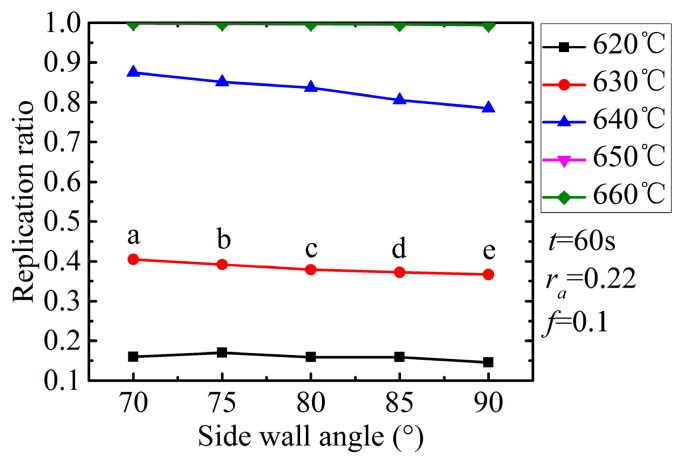
Influence of side wall angle on replication ratio.

**Figure 12:**
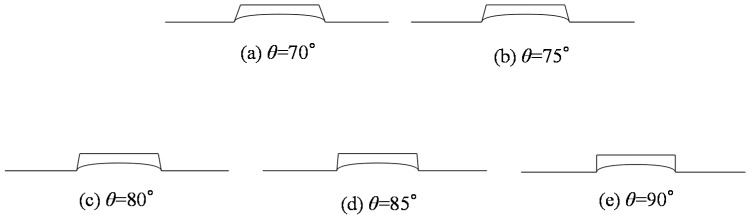
Molded profiles by simulation at different angle degrees (*t* = 60 s, *r*_a_ = 0.22). (**a**) θ = 70°, (**b**) θ = 75°, (**c**) θ = 80°, (**d**) θ = 85°,and (**e**) θ = 90°.

The influence of the side wall angle on the replication ratio is shown in Figure 11. All the curves witnessed a slight drop when the side wall angle increased from 70° to 90°, except for the two curves which were equal to or above 640 °C due to their complete filling. The pattern can be attributed to the flow resistance as well. The flow in a smaller side wall angle groove is supposed to be confronted with lower resistance. However, the influence is almost negligible compared to the influence of the temperature and aspect ratio. The molded profiles by simulation at the temperature of 640 °C were extracted and are shown in Figure 12 correspondingly. It is apparent that the influence of the side wall angle on the replication ratio is negligible.

## 5. Experimental Results and Verification

In order to verify the simulation results, the corresponding experiment had to be conducted. Basically, there were four factors contributing the deviation between the predicted and experimental results as below:

(1)  Step

The models only simulated the pressing step due to simplification, while the experiment consisted of four steps, i.e., heating, pressing, annealing and cooling.

(2)  Constitutive model

The constitutive model was based on the related reference. Although they shared the same material brand name, the constitutive properties might be different to some extent as well.

(3)  Mold geometry

The mold geometry in the simulation was simplified so the side wall angle was 90° and the corner radius was rounded with a radius of 0.5 µm.

(4)  Glass thickness

The glass thickness in the experiment was around 500 μm, while it was 50 μm in the simulation due to simplification.

Therefore, it is hard to achieve direct quantitative verification at this stage. In this paper, the qualitative and indirect quantitative verification were conducted.

**Figure 13:**
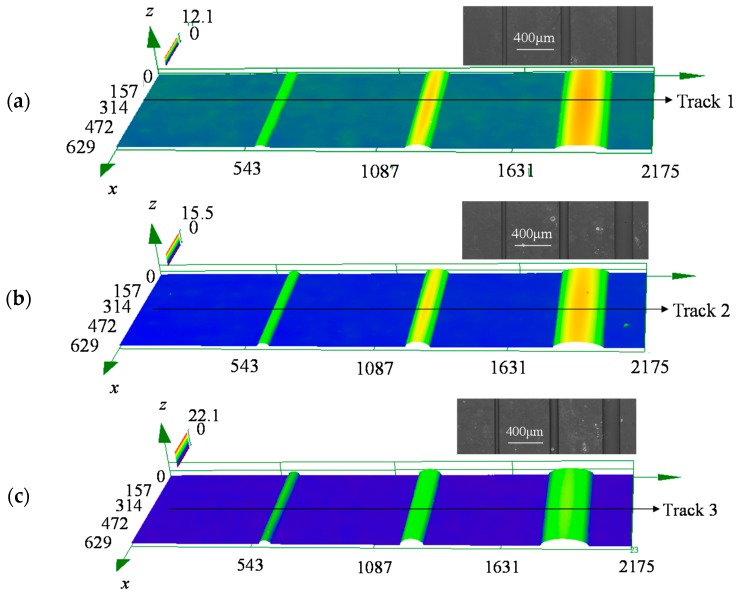
Three dimensional (3D) morphology of molded preform (press time 60 s, pressures 0.3 MPa. (**a**) *T* = 620 °C, (**b**) *T* = 630 °C and (**c**) *T* = 640 °C.

Figure 13 shows the 3D morphology of the molded preform pressed at different temperatures, i.e., 620, 630 and 640 °C. A high temperature can lead to the high flowability of the preform, which makes it easy to deform. Comparing the three photos, it is obvious that the higher the pressing temperature, the higher the swell height. The maximum height was 12.1 μm at 620 °C, while it changed to 15.5 and 22.1 μm at 630 and 640 °C, respectively. The trend is consistent with the results in Figure 7. In addition, when only one photo was analyzed, it is obvious that the wider the groove, the higher the swell height. The trend is consistent with the results in Figure 9.

**Figure 14:**
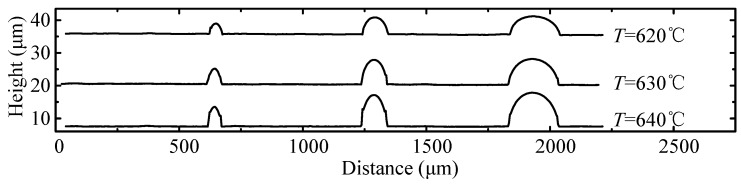
The two dimensional (2D) profiles of the molded preform.

**Figure 15:**
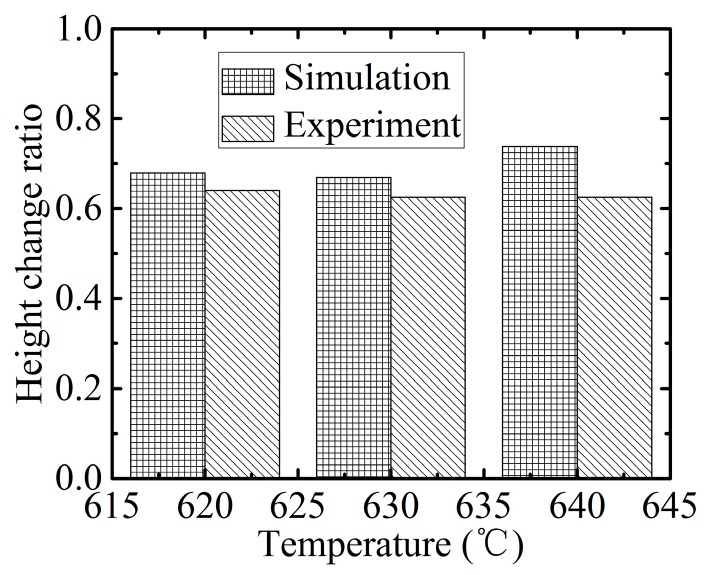
The height change ratio versus aspect ratio.

In order to provide more details of the molded preform, a track line is drawn in each photo in Figure 13, and the corresponding 2D profiles are shown in Figure 14. All molded shapes are similar to the simulation result in that they appear to be upward salient arcs before achieving the full filled condition. To evaluate the change in the replication ratio versus the aspect ratio quantitatively, their heights in Figure 15 are recorded, and the height change rate was defined as the height of the aspect ratio 0.44 divided by that of 0.11. Figure 15 demonstrates the height change ratio versus the aspect ratio. It indicates that there is an obvious influence of the aspect ratio on the swell height. Specifically, the height generated at the aspect ratio of 0.44 was around 65% of that at the aspect ratio of 0.11. The deviation error between the simulation and experimental results is around 10%, although the height change ratio is higher from predicted results. Therefore, the simulation results are verified by the experimental results successfully, and it proves again that the change in aspect ratio has an important influence on the replication ratio.

## 6. Conclusions

The paper investigated the formation process of cuboid microprotrusions by the glass molding process, and the following conclusions can be made:

(1) The maximum stress was located around the bottom corner of the top mold. High stress was generated when the glass contacted the ceiling and side wall of the mold, and it gradually approached the unfilled corner when the molding time was increased further.
(2) The replication ratio dramatically increased with the pressing temperature, while the pattern was reversed with the increase of the aspect ratio. The influence of the angle degree was negligible, though some slight decrease of the replication ratio with the increase of the degree angle was witnessed.
(3) The results from GMP experiments confirmed that the change in the aspect ratio had an important influence on the replication ratio. By comparing the experimental and predicted results, the established FEM model was verified.

## Figures and Tables

**Table 1:** The thermal and mechanical properties of soda-lime preform. Data from [18].

Property	Value
Module of elasticity, *E* (GPa)	62
Module of rigidity, *G* (GPa)	25.4
Possion’s Ratio, υ	0.22
Density, ρ (kg/m^3^)	2500
Specific Heat, cp (J/(kg·K))	880
Thermal Conductivity, κ (W/(m·K))	0.937
Glass Transition Temperature, *T*_g_ (°C)	550
Softened temperature, *T*_s_ (°C)	720

**Table 2:** The parameters of the Generalized Maxwell model. Data from [19].

Term No.	Reduced Time λ_i_ (s)	Relative Moduli ω_i_
1	8.0790 × 10^−5^	5.52214 × 10^−2^
2	1.4580 × 10^−3^	8.20598 × 10^−2^
3	1.8460 × 10^−2^	1.21502 × 10^−1^
4	2.0380 × 10^−1^	2.28594 × 10^−1^
5	9.1391 × 10^−1^	2.86077 × 10^−1^
6	4.0130	2.26545 × 10^−1^
